# Folding Mechanism and Aggregation Propensity of the KH0 Domain of FMRP and Its R138Q Pathological Variant

**DOI:** 10.3390/ijms232012178

**Published:** 2022-10-12

**Authors:** Daniele Santorelli, Francesca Troilo, Francesca Fata, Francesco Angelucci, Nicola Demitri, Giorgio Giardina, Luca Federici, Flavia Catalano, Adele Di Matteo, Carlo Travaglini-Allocatelli

**Affiliations:** 1 Department of Biochemical Sciences “A Rossi Fanelli”—Sapienza, University of Rome, 00185 Rome, Italy; 2 Institute of Molecular Biology and Pathology National Research Council of Italy, 00185 Rome, Italy; 3 Department of Health, Life and Environmental Sciences, University of L’Aquila, 67100 L’Aquila, Italy; 4 Elettra—Sincrotrone Trieste, S.S. 14 Km 163.5, Area Science Park, Basovizza, 34149 Trieste, Italy; 5 Department of Innovative Technologies in Medicine and Dentistry and Center for Advanced Studies and Technology (CAST), University of Chieti “G. d’Annunzio”, 66100 Chieti, Italy

**Keywords:** FMRP, KH domains, folding mechanism, folding intermediate, amyloid fibrils

## Abstract

The K-homology (KH) domains are small, structurally conserved domains found in proteins of different origins characterized by a central conserved βααβ “core” and a GxxG motif in the loop between the two helices of the KH core. In the eukaryotic KHI type, additional αβ elements decorate the “core” at the C-terminus. Proteins containing KH domains perform different functions and several diseases have been associated with mutations in these domains, including those in the fragile X mental retardation protein (FMRP). FMRP is an RNA-binding protein crucial for the control of RNA metabolism whose lack or mutations lead to fragile X syndrome (FXS). Among missense mutations, the R138Q substitution is in the KH0 degenerated domain lacking the classical GxxG motif. By combining equilibrium and kinetic experiments, we present a characterization of the folding mechanism of the KH0 domain from the FMRP wild-type and of the R138Q variant showing that in both cases the folding mechanism implies the accumulation of an on-pathway transient intermediate. Moreover, by exploiting a battery of biophysical techniques, we show that the KH0 domain has the propensity to form amyloid-like aggregates in mild conditions in vitro and that the R138Q mutation leads to a general destabilization of the protein and to an increased fibrillogenesis propensity.

## 1. Introduction

The K-homology (KH) domain is a small, structurally conserved domain originally identified in the human heterogeneous nuclear ribonucleoprotein K (hnRNP K) [1,2]. This domain has been found in single or multi-copies (organized separately or forming a two-domain structural unit) in proteins from archaea, bacteria, and eukaryotes and is generally devoted to RNA or ssDNA recognition [2,3].

KH domains share low sequence homology but have a well-conserved three-dimensional structure consisting of: (i) α helices and β strands elements arranged in a central βααβ “core” (the KH “core”); (ii) a conserved (I/L/V)IGxxGxx (I/L/V) motif, located between the two helices of the “core”, which has been proposed to mediate nucleic-acid recognition [4]; and (iii) a variable loop [2,3,4,5]. Additional αβ elements flanking the KH “core” either at its N-terminus or C-terminus distinguish eukaryotic type I KH domains (KHI, in which the order of secondary structure elements is βααβ**βα**, prokaryotic type II KH (KHII, **αβ**βααβ) or type III KH, where the two additional elements are split between the N-terminus (the α-helix) and C-terminus (the β-strand)) [2,5]. The overall structure of all KH domains consists therefore of three α-helices packed against a three-stranded β-sheet, but KHI, KHII, and KHIII adopt different topologies [5]. Moreover, some KH domains defined as non-canonical (or degenerate), lack the conserved GxxG motif in the loop connecting the two alpha-helices of the “core”. Non-canonical KH domains have been identified in both eukaryotic and prokaryotic proteins, such as the bacterial ribosome assembly factor RbfA [6], the MTH1203 from the archaeon *M. thermautotrophicus* [7], the Drosophila P-element somatic inhibitor protein (PSI) [8], in proteins belonging to the vigilin family [9], the Rrp40 exosome subunit [10], and in the fragile X mental retardation protein (FMRP) [11]. As degenerated KH domains lack the characteristic GxxG loop, they are difficult to predict from sequence analysis and often can be identified only after structure determination; thus, the list of proteins containing this domain will likely increase over time. 

**Figure 1:**
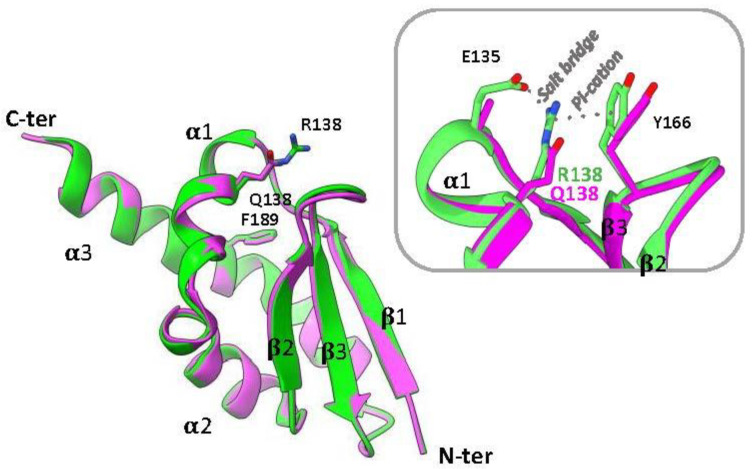
Superposition of the three-dimensional structure of the KH0 (green) and KH0-R138Q domain (magenta) of FMRP represented as a cartoon (from pdb codes 4QVZ and 4QW2, respectively). The residues Arg138/Gln138 and Phe189 are shown in stick representation. Secondary structure elements are labeled. The blow-up shows that Arg138 engages in a salt bridge with Glu135 and makes a Pi-cation interaction with Tyr166.

Proteins containing KH domains perform different functions and several diseases have been associated with mutations in these domains [2,3], including those of the fragile X mental retardation protein (FMRP) [12]. FMRP is involved in cognitive development and female reproductive function [12,13,14]; moreover, its ability to bind many different mRNAs in the brain makes it a key component of RNA granules [15,16,17]. It is a large multidomain protein consisting of three regions: i) the N-terminal region formed by two Tudor domains and one degenerated KHI domain (KH0); ii) a central region with two adjacent KHI domains (KH1 and KH2); and iii) a long C-terminal unstructured region containing arginine- and glycine-rich (RGG) motifs; however, the 3D structure of the entire protein is still lacking [11,12,18,19,20]. The fragile X syndrome (FXS), the most common form of inherited intellectual disability (ID), is typically caused by an expansion of the CGG trinucleotide repeat in the non-coding 5′ untranslated region of the FMR1 gene, leading to hypermethylation of the genes, transcriptional silencing, and thus loss of the FMRP protein. However, FXS can also be caused by missense or nonsense mutations in the protein-coding sequence [14,21,22,23,24]. In particular, the missense mutation R138Q, located in the KH0 domain of FMRP, was found in patients with the FXS typical phenotype [22,23,24,25,26,27]. Interestingly, although this mutation does not affect the 3D structure of the protein [11] (Figure 1), this variant affects the recognition and binding to the BK channels [27] and has been associated with impaired AMPAR-mediated plasticity and socio-cognitive defects in mouse models [28].

Despite the abundance of structural data available on KH domains, and given their crucial functional roles in many proteins [2], it is surprising that the characterization of their dynamic and folding properties is still limited. To our knowledge, although there were some preliminary observations [29], the folding mechanism and aggregation propensity of a KH domain have been studied only recently. In particular, the folding mechanism of RbfA, a KHII-containing protein from *P. aeruginosa*, was shown to fold according to a three-state mechanism involving a transiently populated intermediate and to form fibrils even in mild conditions [6].

Here, we present a characterization of the folding mechanism of the degenerated KH0 domain from the human FMRP protein by equilibrium and kinetic experiments. Since the R138Q mutation does not significantly alter the 3D structure of the domain [11] but affects the FMRP function [26,28], we resorted to investigating this variant by studying its thermodynamic stability and (mis)folding properties in comparison with the wild-type domain. By exploiting a battery of biophysical techniques, we show that the KH0 domain has the propensity to form amyloid-like aggregates and that the R138Q mutation destabilizes the domain and promotes its intrinsic aggregation properties.

## 2. Results

### 2.1. Equilibrium Experiments

The KH0 domain from the FMRP protein (hereafter KH0, res: 122–205) and its R138Q variant (KH0-R138Q) (Figure 1) were produced and purified as reported in the Methods section. Far-UV CD spectra confirm that the purified proteins are correctly folded and the R138Q mutation does not alter the overall structure of the protein (Figure S1), in agreement with the X-ray crystal structure reported [11].

**Figure 2:**
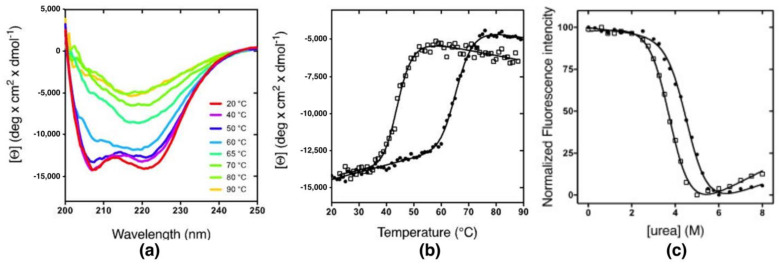
(**a**) Far-UV CD spectra recorded at different temperatures of KH0 measured in 20 mM sodium phosphate pH 7.2, 100 mM NaCl. (**b**) CD thermal denaturation profile of KH0 (full circles) and KH0-R138Q (empty squares) monitored by CD at 222 nm. The curves, fitted to a sigmoidal function, provided an app-T_m_ of 68 ± 1 °C and 48 ± 2 °C for KH0 and KH0-R138Q, respectively. (**c**) Urea-induced denaturation of KH0 (full circles) and KH0-R138Q (empty squares). Fitting of the reversible transitions (black lines) provided the thermodynamic stability (ΔG_DN_) of the two proteins (ΔG_DN_ = 5.7 ± 0.3 and 4.7 ± 0.2 kcal mol^−1^ for KH0 and KH0-R138Q, respectively). The *m* value was shared during fitting procedure for both proteins (*m* = 1.25 ± 0.06 kcal mol^−1^ M^−1^).

To investigate whether the Arg to Gln mutation could affect the stability of the domain, thermal-induced denaturation experiments were carried out on both KH0 and KH0-R138Q. Interestingly, the far-UV CD spectra of KH0 collected at different temperatures revealed the appearance of a single, broad negative band centered around 215 nm above 60 °C, suggesting a thermal-induced transition towards a β-rich structure rather than a denaturation process (Figure 2a); the same behavior was also observed with the R138Q variant (Figure S2). Figure 2b shows the temperature-induced transition of KH0 and KH0-R138Q monitored at 222 nm. Although these processes are cooperative, indicating a transition between two species, they were both irreversible (Figure S3), thus preventing a reliable thermodynamic analysis. A fit to a sigmoidal equation returned the apparent thermal midpoints (app-T_m_) of 68 °C and 48 °C for KH0 and KH0-R138Q, respectively. To properly determine the thermodynamic stability of the two proteins, we carried out urea-induced denaturation experiments. The reversible denaturation transitions monitored by far-UV CD spectroscopy (Figure S4) allowed obtaining the ΔG_DN_ for the two proteins (ΔG_DN_ = 5.5 ± 0.20 and 4.46 ± 0.23 kcal mol^−1^ for KH0 and KH0-R138Q, respectively) (Table 1), highlighting the destabilization of KH0-R138Q by approximately 1 kcal mol^−1^.

Next, to investigate the folding mechanism by fluorescence-monitored (un)folding kinetics, we took advantage of the Trp-containing fluorescent variants of KH0 and KH0-R138Q (hereafter W-KH0 and W-KH0-R138Q, respectively), obtained by replacing the F189 with a Trp residue (Figure 1). Figure 2c shows the reversible and cooperative urea-induced denaturation profiles of W-KH0 and W-KH0-R138Q, suggesting the absence of stable equilibrium intermediate(s). The unfolding free energy (ΔG_DN_) changes, derived from two-state analysis (ΔG_DN_ = 5.7 ± 0.3 kcal mol^−1^ and 4.7 ± 0.2 kcal mol^−1^ for W-KH0 and W-KH0-R138Q, respectively; Table 1) confirm the destabilization of the R138Q variant monitored by far-UV CD experiments. Notably, for wt and R138Q the CD- and fluorescence-monitored denaturation profiles could be reliably fitted sharing the m_DN_ values, in accordance with the observation that the R to Q substitution does not significantly alter the overall KH0 structure [11].

### 2.2. The Folding Mechanism of KH0 and KH0-R138Q

**Figure 3:**
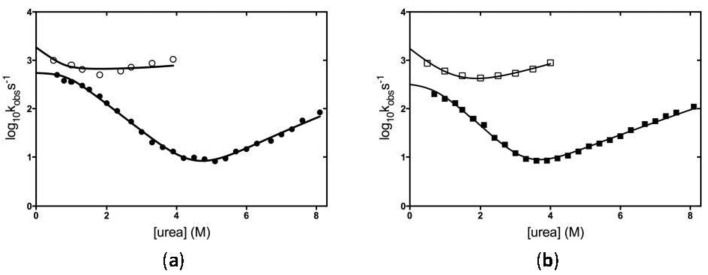
Chevron plot of the observed (un)folding rate constants as a function of urea concentration determined by SF (full symbols) or TJ experiments (empty symbols) for (**a**) W-KH0 and (**b**) W-KH0-R138Q . The solid black line represents the fit to a three-state model with an on-pathway intermediate.

The folding mechanism of the KH0 domain of FMRP was investigated by rapid kinetics experiments. The dependence of the observed folding rate constants (k_obs_) on urea concentration obtained by stopped-flow experiments (SF; closed symbols) and T-jump (TJ; open symbols) are reported in Figure 3a,b for W-KH0 and W-KH0-R138Q, respectively. 

Both proteins show a non-linear dependence of the folding rate constants obtained by SF at low urea concentrations ([urea] < 2 M), an observation that is commonly interpreted as evidence for the transient accumulation of an intermediate during the folding process [30,31]. This observation was further corroborated by the analysis of the amplitudes of the corresponding (un)folding traces (Figure S5). The existence of such an additional and faster folding process, inaccessible by SF experiments, was confirmed by ultra-rapid TJ (un)folding experiments in matching temperature conditions (Figure S6) allowing us to monitor very fast reactions. The global fit of all the rates and associated m-values from SF and TJ experiments (Figure 3) to a three-state mechanism allowed the determination of all the four rate constants implied by the model (Table 2). However, the identification of a folding intermediate is not sufficient per se to distinguish between two alternative three-state folding mechanisms whereby the intermediate may represent an obligatory species along the folding process (on-pathway mechanism) or a kinetic trap in an off-pathway mechanism (D ⇌ I ⇌ N and I ⇌ D ⇌ N, respectively) [31]. To distinguish between these two alternative scenarios, the complete (un)folding kinetics of W-KH0 and W-KH0-R138Q were fitted to both models. The parameters obtained by the on-pathway model (continuous lines in Figure 3 and Table 2) are more satisfactory than those obtained from the off-pathway model, which results in larger errors in the microscopic rate constants and unrealistic m-values (not shown). Over-and-above confirming the destabilization of the N state of the W-KH0-R138Q variant with respect to the W-KH0 calculated from equilibrium experiments (ΔΔG_ND_ approx. 1 kcal/mol; Table 1 and Table 2), the determination of the microscopic rate constants associated to the transition from the intermediate (I) to the native (N) state in the two proteins (k_IN_ = 584 s^−1^ and 243 s^−1^, for W-KH0 and W-KH0-R138Q, respectively; Table 2) allowed us to estimate that the energy barrier between I and N, is higher in the variant protein. Such an observation may have some implications on the greater fibrillogenesis propensity of W-KH0-R138Q (see below).

### 2.3. Aggregation and Fibrillogenesis Propensity of KH0 and KH0-R138Q

Different studies emphasized the relevance of partially folded states in the aggregation process and in amyloid fibril formation [32,33,34,35]. In the case of the KH0 domain from FMRP, we were initially prompted to explore the propensity of KH0 and KH0-R138Q to form fibrillar-like aggregates in vitro by the results of (i) the far-UV CD spectroscopy experiments, which clearly showed an irreversible conformational transition from the N state to β-rich species as a function of temperature and (ii) the observation that the KH0 domain, both the wt and the R138Q variant, fold through a three-state mechanism, implying the formation of a transient intermediate.

**Figure 4:**
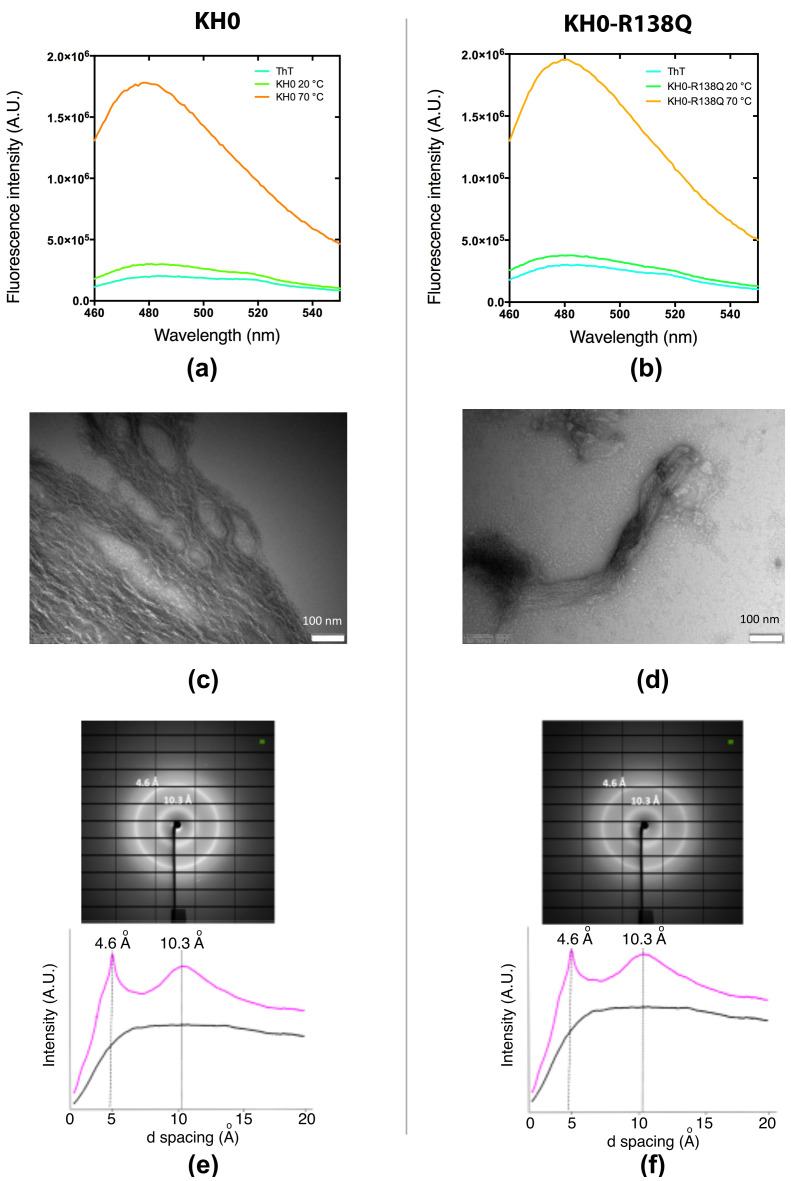
Fluorescence spectra recorded after adding a stoichiometric amount of ThT in pre-incubated samples at 70 °C and 20 °C of 20 μM KH0 (**a**) and KH0-R138Q (**b**) in 20 mM sodium phosphate pH 7.2 100 mM NaCl. TEM analysis. Transmission electron micrograph of heat-induced fibrils after 2 h incubation at 55 °C of 50 μM KH0 (**c**) and 50 μM KH0-R138Q (**d**) The scale bars correspond to 100 nm in both panels. X-ray analysis. X-ray diffraction analysis of KH0 (**e**) and KH0-R138Q (**f**); samples (100 μM) were heated at 37 °C for 50 h.

Thioflavin T (ThT)-binding assays [36,37] were used to detect the formation of fibril-like aggregates at appropriate conditions. KH0 and KH0-R138Q domains (20 μM) pre-incubated at 70 °C for 10 min indeed showed a strong increase of the ThT fluorescence emission at 482 nm, suggesting the formation of fibrillar aggregates under these conditions (Figure 4a,b).

The morphological properties of these fibril-like aggregates were then investigated by TEM and X-ray experiments. KH0 and KH0-R138Q (50 μM) were pre-heated at 55 °C for 2 h in a water bath. As shown in Figure 4c,d, the TEM micrographs showed the presence of fibrillar-like aggregates with diameters between 5 and 15 nm, in accordance with previous reports [6,29,38]. The fibrils of the KH0 sample appeared dense and curved, with an apparent constant diameter and variable lengths (Figure 4c). Interestingly, the fibrils formed by the KH0-R138Q, albeit having similar diameter distribution, are shorter and straighter than those of the wt protein and seem to assemble in larger bundles (Figure 4d). 

To confirm the fibrillar nature of the aggregated samples suggested by ThT and TEM experiments, we performed X-ray diffraction analysis of both KH0 and variant samples (100 μM) heated at 70 °C for 10 min. The diffraction images of both proteins showed the cross-β fiber diffraction pattern typical of amyloid fibrils [39]. To investigate whether the same characteristics could be also obtained at physiological temperature, we performed the same experiments incubating KH0 and KH0-R138Q at 37 °C for 50 h. Figure 4e-f shows that also in these conditions, typical cross-β diffraction patterns were obtained. Indeed, analysis of the diffraction images highlights the presence of a sharp peak at 4.6 Å spacing, indicating the inter-strand distance within β-sheets and a broad peak centered at 10.3 Å, indicating the inter-sheet spacing within the fibril cross-section [39].

**Figure 5:**
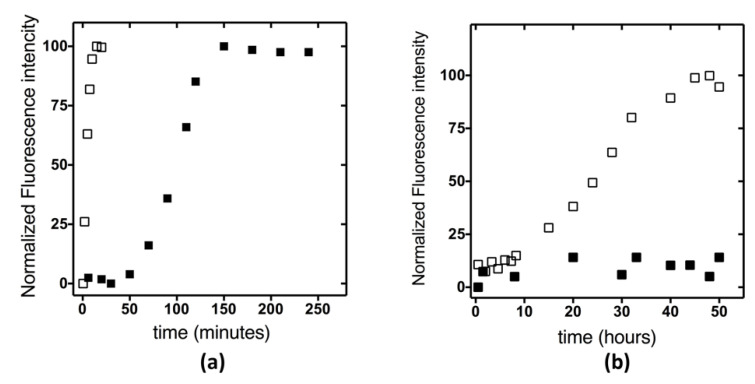
Kinetics of fibril formation followed by ThT fluorescence analysis at 482 nm (λ_ex_ = 440 nm) of KH0 (full squares) and R138Q (empty squares) incubated at 55 °C (**a**) and 37 °C (**b**) in 20 mM sodium phosphate pH 7.2, 100 mM NaCl.

Next, we investigated if the variant protein has a different fibrillar propensity with respect to the wt, analyzing the kinetics of fibril formation by ThT assay (Figure 5). Experiments performed at 55 °C (10 μM proteins concentration) showed that the kinetics of the amyloid fibrils formation for the wt protein occurs with a defined lag phase with a t_1/2_ of approximately 2 h, while in the case of KH0-R138Q, the process is completed in less than 30 min (Figure 5a). Interestingly, for the variant protein, a clear sigmoidal profile with a lag phase could be observed only when the experiment was carried out at the lower incubation temperature (37 °C), with a t_1/2_ of approximately 25 h, while no ThT emission was observed for KH0 in this condition (Figure 5b).

Overall, these results show that the KH0 domain of the FMRP protein has a propensity to form fibrillar-like aggregates in vitro even in mild conditions and, notably, that the R to Q substitution confers to this domain a stronger propensity to form fibrillar aggregates.

## 3. Discussion

**Figure 6:**
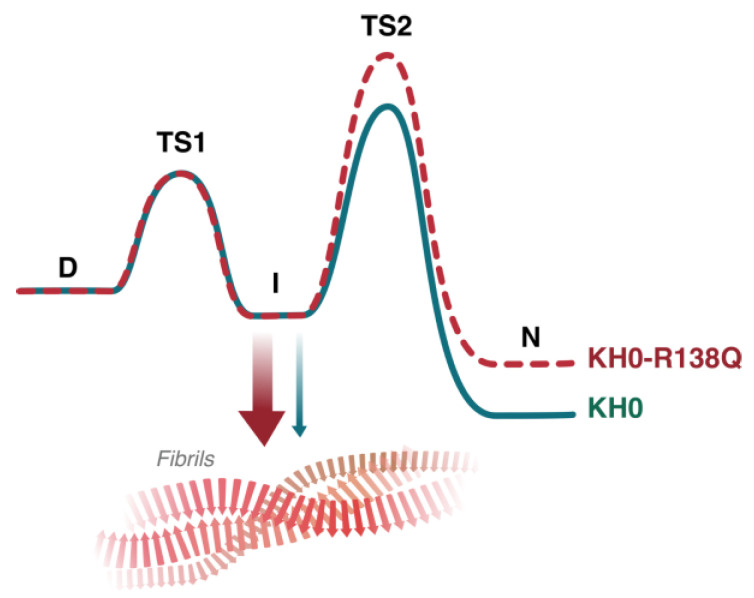
Proposed model for the enhanced aggregation propensity of KH0-R138Q mutant. In the case of KH0-R138Q mutant, a more populated intermediate state (I), due to a higher energy barrier between the I and N state (TS2), could facilitate KH0-R138Q reaching the fibrillar state. It cannot be excluded that the destabilization of the native state of KH0-R138Q with respect to KH0 (N) also plays a role in the fibrillogenesis process.

In the present manuscript, we present the results of biophysical experiments aimed at characterizing the folding mechanism of the KH0 domain of the human FMRP protein and its pathological variant R138Q. This mutation occurs in the α1 helix of the KH0 domain (Figure 1) [11] and has been identified in patients with clinical features of FXS [11,22,24,25]. We have shown that the folding of KH0 proceeds through the transient accumulation of an intermediate state, which, according to the global adaptation of the data of the kinetic experiments of rapid (SF) and ultrarapid (TJ) folding, represents an obligatory species along the folding process. This three-state folding mechanism is conserved in KH0-R138Q, as expected for a protein that maintains the same 3D structure [11] and is reminiscent of the folding mechanism observed for the non-canonical type II KH domain of RbfA [6]. Furthermore, through complementary biochemical techniques, we have shown that KH0 is prone to form fibrillar-like aggregates and that, even if the folding properties of the KH0 domain appear to be maintained in the R138Q variant, the propensity to aggregate of the variant appears to be increased. In fact, the ThT-based analysis showed a marked difference in the kinetics of fibril formation between KH0 and KH0-R138Q, with an evident acceleration of the rate of the fibrillogenesis process in the variant under the tested conditions. Indeed, the midpoint of the thermally induced transition monitored by CD of KH0-R138Q is lower than that of the KH0 wt (ΔT_app_ ≈ 20 °C), further highlighting the enhanced propensity of the mutant to undergo fibrillogenesis. These observations could be interpreted on the basis of the thermodynamic and kinetic parameters of folding presented in this work (Table 1 and Table 2, respectively). Although the stability of the intermediate species is barely affected by the mutation, the N state of KH0-R138Q is relatively destabilized and its formation is under kinetic control since the energy barrier for the transition from I to N is higher in KH0-R138Q. This effect is likely due to the disruption of the interactions of R138 with E135 and Y166 that are not conserved in KH0-R138Q (Figure 1). Assuming that the folding intermediate is responsible for triggering the aggregation process, as demonstrated in many cases [32,33,34], the slower folding process from I to N in the variant protein will result in a higher population of species subject to the aggregation over time and, finally, a more rapid fibrillogenesis process (Figure 6). Future studies aimed at the characterization of the structure of this partially folded intermediate based on phi-value analysis [40,41], together with the possibility to describe the free energy landscape of fibril formation on the basis of molecular dynamics simulation [42,43], will hopefully pave the way to a detailed structural mechanism for the aggregation of KH0 and KH0-R138Q.

Given the low sequence homology and the high structural conservation, KH domains are particularly fascinating models for folding studies [5,44,45]. Future studies aimed at characterizing the process of canonical KH domains of different origins would be interesting to understand if they share a common folding mechanism or not and if differences do exist between canonical and non-canonical KH domains. Over and above the study of the folding process of KH domains, our work stresses the KH0 propensity for the formation of fibrillar aggregates. Indeed, a propensity of the N-terminal half of FMRP to the formation of β-rich structures under physiological conditions was already proposed by Sjekloca and colleagues [29]. Our work, in addition to confirming these data, underlines how the region corresponding to the KH0 has a fundamental role in this process, suggesting a specific function of this domain in protein–protein interaction.

A fascinating hypothesis is that the KH0 domain, thanks to its intrinsic propensity to form fibrillar aggregates, is the portion of FMRP that mostly contributes together with the C-terminal ID to the intermolecular interactions necessary for the formation of different types of FMRP containing RNP granules. Under pathological conditions, the R138Q mutant leads to a general destabilization of the protein and increases its fibrillogenesis tendency. Moreover, since this R to Q mutation does not alter the general structure of the protein but affects a residue on its surface, it also likely influences its interaction with physiological partners, altering two competitive processes such as physiological/toxic hetero- and self-aggregation.

## 4. Materials and Methods

### 4.1. Protein Expression and Purification

The KH0 domain (res: 122–205) of the FMRP (Uniprot: Q06787) was synthetized by GenScript (Piscataway, NJ, USA) and cloned in a pET 28b (+) expression. The KH0-R138Q, KH0-F189W and the KH0-R138Q/F189W were generated by QuickChange Lightning Site-Directed Mutagenesis kit (Agilent technologies, Santa Clara, CA, USA) according to the manufacturer’s instructions.

Proteins were expressed in the *Escherichia Coli* BL-21 (DE3) (BioLabs) strain. Cultures were grown in Luria–Bertani (LB) medium containing 34 μg/mL Kanamycin at 37 °C. After induction with 0.5 mM IPTG (isopropyl-β-d-thiogalactopyranoside), cells were grown at 21 °C over-night and collected by centrifugation. Pellets were resuspended in 20 mM Tris-HCl pH 8.0, 300 mM NaCl, 15 mM imidazole and glycerol 5%, protease inhibitor (Complete EDTA-free, Roche, Basel, CH) and sonicated. The supernatant was loaded on a HisTrap FF (GE Healthcare, Chicago, IL, USA) column equilibrated with the same buffer. Proteins were eluted with an imidazole gradient (20 mM–1 M) and collected fractions were buffer exchanged with 20 mM Tris-HCl pH 8.0, 100 mM NaCl with a HiTrap Desalting column (GE Healthcare).

To perform fibrillation/aggregation experiments the histidine tag was removed by Thrombin (Sigma-Aldrich, St. Louis, MO, USA) cleavage (4U/mg for 2 h at 4 °C) and subsequently purified by size exclusion chromatography (Superdex75 10/300; GE Healthcare).

### 4.2. Equilibrium Experiments

Circular dichroism (CD) experiments were performed in the far-UV region using a Jasco J710 instrument (Jasco Inc., Easton, MD, USA) equipped with a Peltier apparatus. Spectra were collected using 15 μM proteins in 20 mM sodium phosphate pH 7.2, 100 mM NaCl using a 1 mm quartz cell (scanning speed of 100 nm/min, average of three acquisitions). Thermal denaturations were performed by monitoring the CD signal at 222 nm (1 °C/min thermal ramp, from 20 °C to 90 °C). Equilibrium urea-induced denaturations were performed with 25 μM proteins following the change in ellipticity at 222 nm upon urea addition. Intrinsic fluorescence emission measurements were carried out with FluoroMax-4 spectrofluorometer (Jobin Yvon, Edison, NJ, USA) using a 1 cm path length quartz cuvette. Urea-induced denaturations ([proteins] = 6 μM in 20 mM Na phosphate pH 7.2, 100 mM NaCl) at 20 °C were followed by recording the fluorescence emission at 350 nm (λ_ex_ = 280 nm) as a function of the denaturant concentration.

### 4.3. Kinetic (Un)Folding Experiments

(Un)folding kinetics experiments were performed using an SX-18 stopped-flow apparatus (Applied Photophysics, Leatherhead, UK). Fluorescence emission was measured using a 320 nm cut-off glass filter (λ_ex_ = 280 nm). At least 5 individual traces were acquired and then averaged for each experiment. All the averages were satisfactorily fitted with a single exponential equation. Experiments were conducted using of 3 μM (after mixing) protein sample in 20 mM Na phosphate, pH 7.2, 100 mM NaCl and different concentrations of urea, ranging from 0 to 8.1 M, at 20 °C.

The ultra-fast relaxation kinetics were measured by using a Hi-Tech PTJ-64 capacitor-discharge T-jump apparatus (Hi-Tech, Salisbury, UK). Degassed and filtered samples (30 μM) were slowly injected through the 0.5 × 2 mm quartz flow cell before data acquisition. Buffer used was 20 mM sodium phosphate pH 7.2, 100 mM NaCl at different [Urea]. Fluorescence emission (λ_ex_ = 296 nm) was measured using a 320 nm cut-off glass filter. Temperature jumps were from 11 °C to 20 °C, and 25 traces were usually averaged. All traces were satisfactorily fitted to a mono-exponential decay model.

### 4.4. Thioflavin T Fluorescence Assay

Thioflavin T (ThT; Sigma-Aldrich) stock solution (2.5 mM) was added to proteins pre-incubated for 10 min at 20 °C or 70 °C (final [ThT] and [proteins] = 20 μM). ThT fluorescence spectra were recorded between 460 and 550 nm (λ_ex_ = 440 nm) with a FluoroMax-4 spectrofluorometer (Jobin Yvon, Edison, NJ, USA) using a 1 cm path-length quartz cuvette in 20 mM sodium phosphate pH 7.2, 100 mM NaCl. Aggregation kinetic experiments at different temperatures were carried out in the same conditions by adding a stoichiometric amount of ThT to a KH0 or KH0-R138Q (10 μM) preincubated at 37 °C or 55 °C. The kinetics were followed by recording fluorescence spectra over time and then plotting the emission at 482 nm as a function of time.

### 4.5. TEM Experiments

The KH0 and KH0-R138Q samples were centrifuged for 5 min at 10,000 rpm and diluted with 20 mM Tris-HCl pH 8.0, 100 mM NaCl, to 50 μM. Solutions of both proteins were incubated in a water bath for 2 h at 55 °C. Samples were further diluted to 0.1 mg/mL using the same buffer for the preparation of the grids. 10 μL of each sample was absorbed for 30 s on a 200-mesh carbon-coated copper grid, the excess of sample removed and after a washing step with deionized water negatively stained for 30 s with a filtered solution of freshly prepared 2% uranyl acetate. After a second wash step and air drying, the grids were analyzed at 100 kV using a Philips CM 100 transmission electron microscope (TEM), provided with a tungsten hexaboride filament and a Phurona (Emsis) CMOS camera for image acquisition. The images were processed using ImageJ 1.53k to measure fibril dimensions and to subtract the noisy background [46].

### 4.6. X-ray Diffraction

Diffraction experiments were carried out at the XRD2 beamline at Elettra synchrotron, Trieste (Italy) [47]. Data were collected at room temperature using a monochromatic wavelength of 1.000 Å on a Pilatus 6M hybrid-pixel area detector (DECTRIS Ltd., Baden-Daettwil, Switzerland) at a working distance of 400 mm and a circular beam size of 100 µm^2^ diameter. 

KH0 and KH0-R138Q (100 μM) were heated at 70 °C for 10 min or 37 °C for 50 h, centrifugated, and then pellets were washed three times with MQ water to remove buffer residues. Kapton mesh loops (MiTeGen, Ithaca, NY, USA) were used as support to let 10 μL protein aliquots run dry. To collect diffraction images, the resulting dried thin layer of randomly oriented fibrils was characterized without spinning, using an exposure time of 300 s. One-dimensional diffraction patterns were obtained by radial integration of the area detector images using FIT2D program [48].

Standard experimental setup calibration was performed using a capillary filled with lanthanum hexaboride (LaB6-NIST 660a) powder [49].

### 4.7. Data Analysis

All urea-induced equilibrium unfolding transitions monitored by far-UV CD ellipticity or intrinsic fluorescence emission were analyzed by fitting the baseline and transition region data to a two-state linear extrapolation model [50] according to the following:

ΔG_Unf_ = ΔG^H2O^ + m [Urea] – RTln(K_Unf_)

where ΔG_Unf_ is the free energy change for unfolding for a given denaturant concertation, ΔG^H2O^ the free energy change for unfolding in the absence of denaturant and m a term which quantifies the change in ΔG_Unf_ as function of denaturant, R the gas constant, T the temperature (K), and K_Unf_ the equilibrium constant for unfolding.

Kinetic data were analyzed on the basis of a three-state model, with either an on- or off-pathway intermediate, assuming a linear dependence of the logarithm of the microscopic rate constants on the denaturant concentration [31].

## Figures and Tables

**Table 1:** Urea-induced thermodynamic parameters obtained by fluorescence and circular dichroism equilibrium experiments. The *m* parameter was shared between the data sets of the non-fluorescent variants in the CD-monitored denaturations, between the fluorescent variants in the fluorescence-monitored denaturations and between the data sets of the fluorescent variants in the fluorescence-monitored denaturations. [Urea]_1/2_ in M; *m* in kcal mol^−1^ M^−1^; ΔG_D-N_ in kcal mol^−1^).

Circular Dichroism			Fluorescence	
	KH0	KH0-R138Q	W-KH0	W-KH0 R138Q
**[Urea]_1/2_**	4.30 ± 0.04	3.46 ± 0.06	4.56 ± 0.04	3.77 ± 0.04
***m***	1.29 ± 0.06	1.29 ± 0.06	1.25 ± 0.06	1.25 ± 0.06
**DG_D-N_**	5.50 ± 0.20	4.46 ± 0.23	5.70 ± 0.32	4.70 ± 0.24

**Table 2:** (Un)folding kinetics parameters obtained for W-KH0 and W-R138Q-KH0 according to 3-state model with an on-pathway intermediate (D ⇌ I ⇌ N). Rate constants (k_IJ_) are expressed in s^−1^ and the associated *m* values (*m*_IJ_) are expressed in kcal mol^−1^ M^−1^).

	W-KH0	W-KH0 R138Q
**k_DI_**	1860 ± 220	1723 ± 123
***m*** **_DI_**	0.75 ± 0.04	0.87 ± 0.02
**k_ID_**	29 ± 20	54 ± 6
***m_ID_***	0.22 ± 0.10	0.33 ± 0.03
**k_IN_**	584 ± 58	243 ± 12
***m_IN_***	10^−12^ ± 10^−3^	10^−12^ ± 10^−3^
**k_NI_**	1.62 ± 1.1	1.20 ± 0.04
***m_NI_***	0.32 ± 0.06	0.32 (fixed)

## Supplementary Materials

The following supporting information can be downloaded at: https://www.mdpi.com/article/10.3390/ijms232012178/s1.
