# Supplementary material for: Folding Mechanism and Aggregation Propensity of the KH0 Domain of FMRP and Its R138Q Pathological Variant
Source: Int J Mol Sci. 2022 Oct 12;23(20):12178. doi: 10.3390/ijms232012178 (PMC9603430; doi:10.3390/ijms232012178)
Supplement: Supplementary file 1 [file ijms-23-12178-s001.zip › ijms-1942851-supplementary.pdf]

Supplementary Materials

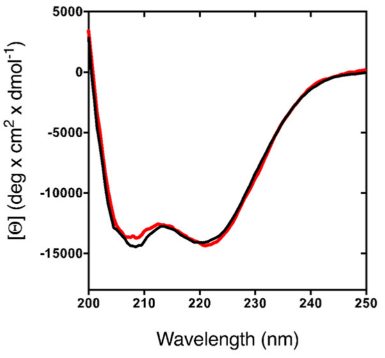

Figure S1. Far-UV CD spectra of KH0 (black line) and KH0-R138Q (red line).

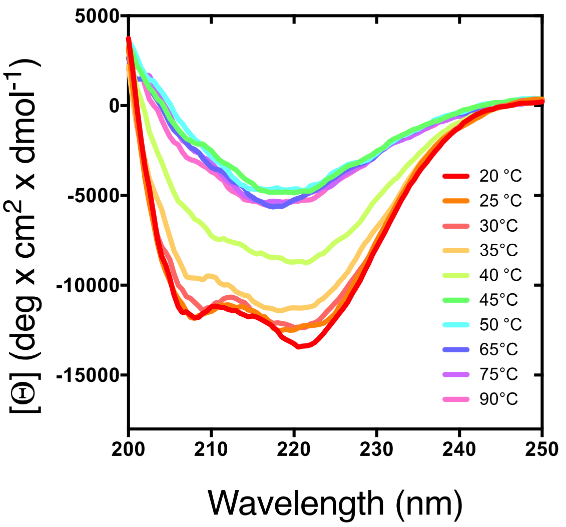

Figure S2. Far-UV CD spectra of KH0-R138Q in 20 mM sodium phosphate pH 7.2, 100 mM NaCl recorded at different temperatures.

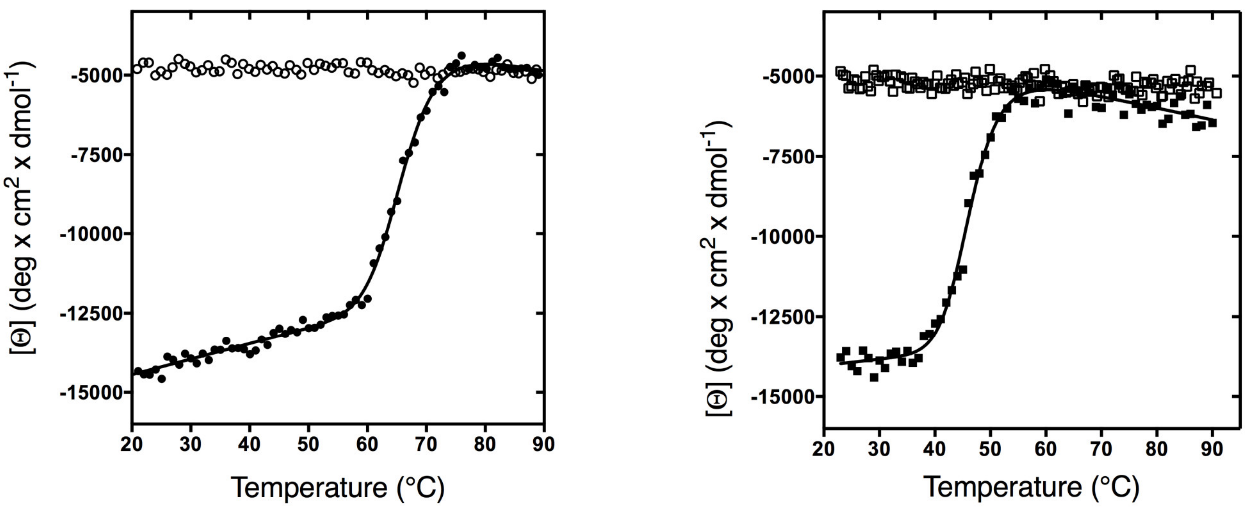

Figure S3. CD thermal denaturation profile (filled symbols) and reverse process (empty symbols) of KH0 (left panel) and KH0-R138Q (right panel) monitored by CD at 222 nm.

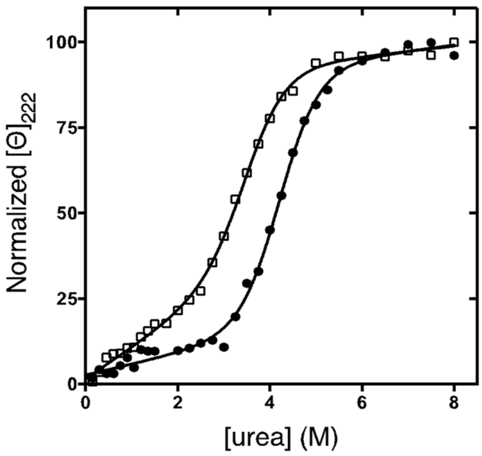

Figure S4. Urea-induced chemical denaturation of KH0 (Full circles) and KH0-R138Q (Empty squares) followed at 222 nm. The line is the best fit to a two-state model, sharing the *m* values.

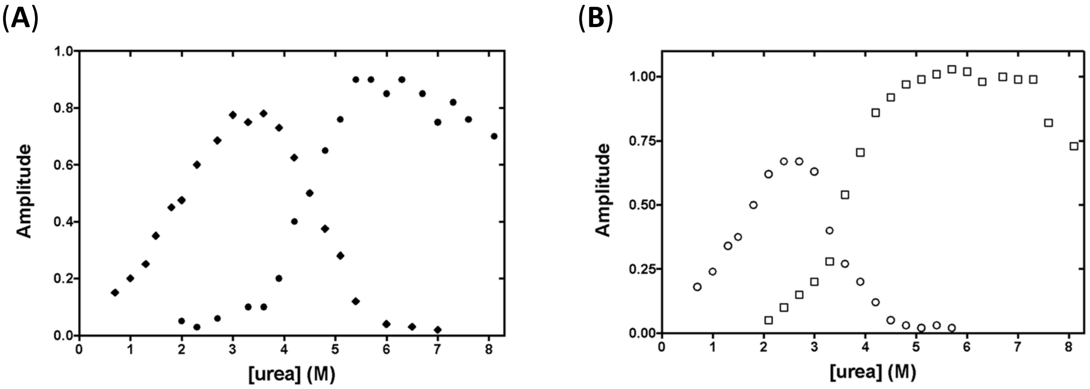

Figure S5. Urea dependencies of the fluorescence amplitudes for (**A**) W-KH0 refolding (full diamonds) and unfolding (full circles) and (**B**) W-KH0-R138Q refolding (empty circles) and unfolding (empty squares).

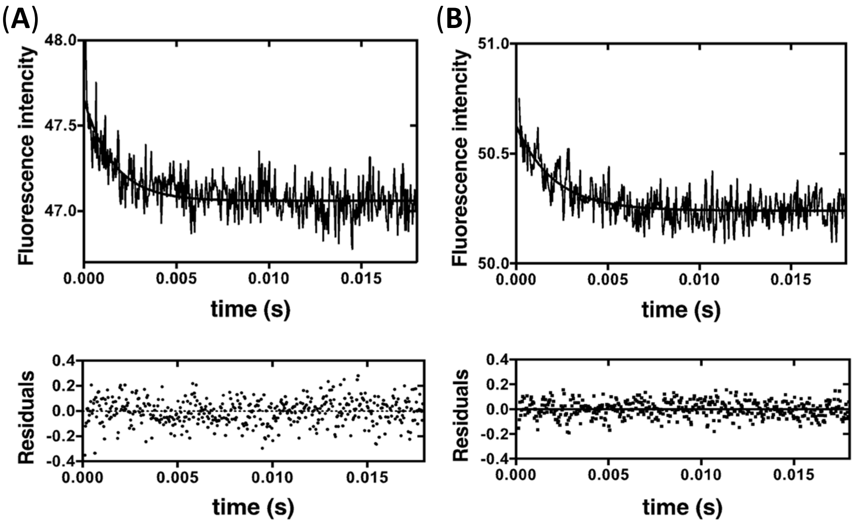

Figure S6. Representative T-Jump time courses recorded at 2,4 M urea for W-KH0 (**A**), and 1,5 M urea for W-KH0-R138Q (**B**), both fitted with a single exponential decay. The bottom panels display the relative residuals plots.
